# “Friends and foes” of multiple myeloma measurable/minimal residual disease evaluation by next generation flow

**DOI:** 10.3389/fonc.2022.1057713

**Published:** 2022-11-28

**Authors:** Paola Pacelli, Donatella Raspadori, Elena Bestoso, Alessandro Gozzetti, Monica Bocchia

**Affiliations:** ^1^ Hematology Unit, Department of Medicine, Surgery and Neuroscience, University of Siena, Siena, Italy; ^2^ Hematology Unit, Siena University Hospital, Siena, Italy

**Keywords:** multiple myeloma, minimal residual disease, next generation flow, complete remission, progression free survival

## Abstract

Next Generation Flow (NGF) represents a gold standard for the evaluation of Minimal Residual Disease (MRD) in Multiple Myeloma (MM) patients at any stage of treatment. Although the assessment of MRD is still not universally employed in clinical practice, numerous studies have demonstrated the strength of MRD as a reliable predictor of long-term outcome, and its potential to supersede the prognostic value of CR. The possibility to acquire millions of events, in combination with the use of standard reagents and a good expertise in the analysis of rare populations, led to high chance of success and a sensitivity of 10^-6^ that is superimposable to the one of Next Generation Sequencing molecular techniques. Some minor bias, correlated to the protocols applied, to the quality of samples and to the high heterogeneity of plasma cells phenotype, may be overcome using standard protocols and having at disposition personnel expertise for MRD analysis. With the use of NGF we can today enter a new phase of the quantification of residual disease, switching from the definition of “minimal” residual disease to “measurable” residual disease. This review takes account of the principle “friends and foes” of Myeloma “Measurable” Residual Disease evaluation by NGF, to give insights into the potentiality of this technique. The optimization of the quality of BM samples and the analytic expertise that permits to discriminate properly the rare pathologic clones, are the keys for obtaining results with a high clinical value that could be of great impact and relevance in the future.

## Introduction

Multiple Myeloma (MM) is a Plasma Cells (PCs) malignancy characterized by the uncontrolled proliferation of pathologic PCs in the Bone Marrow (BM) (1). These cells secrete a monoclonal nonfunctional immunoglobulin (M protein) whose accumulation causes the typical clinical symptoms of the disease, such as hypercalcemia, renal impairment, anemia, and bone lesions (i.e., CRAB criteria) (2–4). MM median age at presentation is above 70 years, and its incidence has increased in the last 25 years, representing today 1-2% of all cancers and about 10% of hematological diseases (5). In recent years, the introduction of new drugs has improved Progression Free Survival (PFS) and Overall Survival (OS) of MM patients (from a median of 3–4 y to a median of 8–9 y) (6, 7). These drugs comprise the immunomodulatory (IMIDs) Thalidomide, Lenalidomide, and Pomalidomide; the Proteasome Inhibitors (PIs) Bortezomib, Carfilzomib, Ixazomib; the Monoclonal Antibodies (MoA) Daratumumab, Elotuzumab, Isatuximab, Belantamab (8). They can be used alone or combined in triplet or quadruplet, leading to exceptional responses that can reach 90% of the treated patients (9), and they are useful to treat also aggressive conditions, such as extramedullary disease (10–12). Moreover, strategies such as consolidation therapy and maintenance after Autologous Stem Cell Transplantation (ASCT) contribute to further improvement of PFS and OS (13–15).

However, in some cases MM patients may still relapse or develop resistance to treatment regimens, leading to the necessity of better and higher-sensitive techniques to monitor Minimal Residual Disease (MRD) and discriminate patients at risk for relapsing. Indeed, the achievement of MRD negativity has superseded the conventional Complete Response (CR) and has been showed as a surrogate endpoint for Progression Free Survival and Overall Survival (16). Clinicians need to deal with MRD assessment in routine clinical practice, and its use in taking therapeutic decisions surely represents one of the most challenging but fascinating issues to be addressed in the next years (17). The evident survival progress and better quality of life of MM patients, associated with higher chances to reach and maintain deep responses, pave the way to the hope that Myeloma could not be anymore an “incurable disease” (18). In this context, Next-Generation Sequencing (NGS) and Multicolor Flow Cytometry (MFC) are currently the best techniques available to monitor MM patients and evaluate MRD with sensitivity up to 10^-6^ (19–22).

### NGS vs NGF

The molecular techniques use the clonal Immunoglobulin (Ig) gene rearrangement as target for the detection of MM MRD levels. The Allele-specific Oligonucleotide Polymerase Chain Reaction (ASO-PCR) and digital PCR (dPCR) have been widely substituted by Next Generation Sequencing (NGS), whose high sensitivity permits to obtain optimal MRD results. However, the feasibility of this approach is limited by high costs, long turnaround time, and required specific expertise (23). Multicolor Flow Cytometry (MCF), on the other hand, is efficient in detecting and quantifying normal vs. pathologic PCs by looking at both markers present on the surface of cells or in the cytoplasm. PCs are characterized by the expression at high level of two main markers, CD38 and CD138; however, MM PCs may be recognized because they could express markers such as CD56, CD28, CD200 and CD117, and, compared with normal PCs, generally are CD45^−^low, CD19^−^, CD27^−^, and CD81^−^.All together, these markers, in addition to the clonal restriction of MM PCs to just one of two immunoglobulin light chains, κ or λ, contribute to easily discriminating normal from clonal MM PCs (24). Older conventional flow cytometric assays are now replaced by advanced assays that permit to simultaneously assess more than eight markers; the great step forward has been made with the introduction of Next Generation Flow (NGF), the high-standardized approach, developed by Flores-Montero et al. (25) which permits, by acquiring ≧̸10^7^ cells, to reach a sensitivity that is indeed superimposable to NGS, but with shorter turnaround time and a substantial costs reduction. Although different combinations of antibodies have been tested, using in-house cocktails, i.e., 10-color (26, 27) or 8-color single-tube (28, 29), the protocol developed by the EuroFlow™ Consortium, has been validated for MRD definition in several studies (30, 31). This protocol, based on the use of two single eight-color tubes containing the markers for MM PCs recognition and combined with the use of specific Standard Operating Procedures (SOPs) that could guarantee the best results in terms of MRD evaluation, has become the gold standard in use in the majority of laboratories. **Table 1** summarizes the characteristics of NGS vs. NGF techniques for MM MRD measurement. The choice of NGS and/or NGF for MM MRD evaluation nowadays just depends on the availability of the laboratory (23, 32, 33), and a hybrid approach, that permits to simultaneously assess MRD by looking at both molecular and cellular characteristics of myeloma clones, could be of great help when appliable (34, 35).

**Table 1:** NGS vs. NGF characteristics for MM MRD measurement.

	ADVANTAGES	DISADVANTAGES	COMMON FEATURES
**NGF**	99% Applicability 2-3 h turnaround time Not require diagnostic sample Intrinsic hemodilution evaluation Gives cells characteristics Wide Availability High Reproducibility Harmonization Lower costs	Requires 2x10^7^ cells Requires fresh samples Does not give molecular characteristics	Qualitative analysis Sensitivity at 10^-6^
**NGS**	Requires 2-3x10^6^ cells Does not require fresh samples	Lower Applicability 90% Long turnaround time (7 days) Requires diagnostic sample No hemodilution evaluation No cell characteristics Limited Availability High costs	Qualitative analysis Sensitivity at 10^-6^

### Depth and timing of MRD

The International Myeloma Working Group (IMWG) defined response criteria in which MRD negativity cut-off was set at 10^-5^ detected either by NGS or NGF. Together with the bone marrow search for monoclonal plasma cells also whole-body imaging such as PET-CT is important to exclude bone focal lesions that could be a disease “reservoir” for relapse. Many trials are now trying to increase MRD sensitivity to 10^-6^ which seems to be a better predictor of PFS (30, 36). Timing of MRD testing is also important and should be at treatment cessation 3 months after autologous stem cell transplant and every 6 months thereafter, at least for 2 years if negativity is achieved. This systematic evaluation could reveal a sustained MRD negativity status that is crucial for long term remission (37).

## Advantages of using NGF

**Figure 1:**
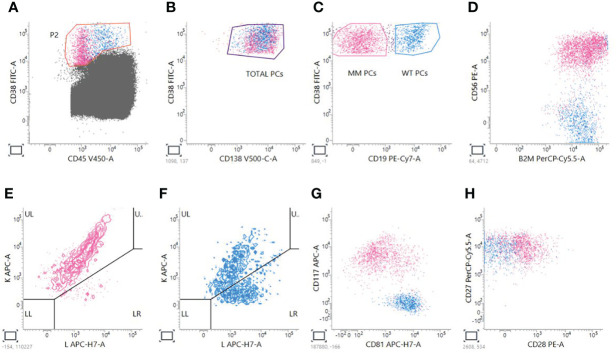
Analysis of MM MRD evaluation by NGF. A sequential gating strategy is used; first of all, PCs are gated on CD38 versus CD45 plot **(A)**; then, CD38^+^CD138^+^ total PCs are taken **(B)**, and two gates are created to distinguish MM CD38^+^CD19^-^ PCs from Wild Type (WT) CD38^+^CD19^+^ PCs **(C)**. For PCST™ tube, we look at expression of CD56 **(D)** and presence of restriction to just one of the two light chains for MM PCs **(E)** compared to WT polyclonal PCs **(F)**. For tube PCD™, we look also at the expression of CD117 and CD81 **(G)**, CD28 and CD27 **(H)**.

Next Generation Flow has many advantages: it is applicable to almost 100% of MM cases, it is very fast, requiring just 2–3 h of processing, and it does not require a diagnostic sample (25). Having at disposition the analysis of the myeloma clone at diagnosis helps defining a pre-treatment panel that could be used as a reference for MRD monitoring (38), as it happens for other leukemias in the so-called Leukemia Associated Immunophenotype (LAIP) approach (39–41). However, it doesn’t overcome the possibility of clonal evolution or the presence of additional subclones that could be minimally represented or be absent at diagnosis, leading to the necessity of considering also a Different from Normal (DfN) approach (42–44). Moreover, in many cases the diagnostic sample is not available for MM patients. In order to obtain the proper Limit Of Detection (LOD), calculated as 50 clonal PCs among 10^7^ nucleated cells, and Limit of Quantification (LOQ), calculated as 20 clonal PCs among 10^7^ nucleated cells, NGF is done by acquiring at least 10 million of events per tube (13, 14); in this way, NGF permits to obtain a high sensitivity of 10^−6^ that is comparable and superimposable to NGS assays (18). The two 8-color pretitred tubes, containing the markers for the recognition of plasma cells, are constructed to perform a sequential gating based on the recognition of the backbone markers (CD45, CD19, CD38, CD138) and the expression of the additional markers that could be aberrantly present on MM PCs surface (CD56, B2, CD117, CD81, CD27, CD28). Moreover, the discrimination of pathologic PCs over the normal counterpart is done by taking into consideration Ig light chains restriction. **Figure 1** shows an example of analysis performed by using a BD Facs Lyric cytometer.

The flow-cytometric assays need to be performed following the Standard Operating Procedures (SOPs), that have been designed by EuroFlow™ to provide full technical standardization and best results for MRD evaluation (25, 45–47); these procedures are applied in order to harmonize reagents, fluorochromes panels, sample processing procedure, platforms used and data analysis (24, 48). Once acquisition of data has been completed by cytometer, subsequent analytic steps are nowadays performed using softwares that permits to merge the data from different analyses and compare the expression of all the markers tested at the different steps of treatment, correlating results with that of MRD data contained in databases. In particular, the Infinicyt™ software developed by EuroFlow™ contains representative flow cytometry data sets from normal healthy BM samples, processed in different standardized centers. These databases are at disposition for the analysis of Acute Leukemias, Chronic Lymphoproliferative Disorders, Primary Immunodeficiencies and Plasma Cells Dyscrasias, and allows for an automated analysis of the complete BM sample, considering both normal and pathologic populations; in this way, the software provides a photograph of the whole immune profile, giving information that may be of great interest and relevant for prognosis of patients and that permit to be confident about MRD results.

MM MRD evaluation is largely performed on Bone Marrow (BM) samples; indeed, BM aspirates are still the gold standard patients’ samples for prognostication and genetic characterization. However, they also represent a limitation due to the aggressiveness of the procedure, to the impossibility, with a single BM aspirate, to reflect the complex MM heterogeneity (15, 49), and to the risk of assessing bad quality BM samples that might not be representative of the real degree of infiltration of the disease. For this reason, recently the same MRD analysis has been tested also on peripheral blood to look at the percentage of Circulating Tumor Cells (CTCs) that could give an idea of patient’s responses to therapy (50, 51). Different studies have already demonstrated the reliability of evaluating CTCs level in MM patients at diagnosis (52) or during different treatment regimens. Detection and isolation of circulating tumor cells (CTCs) is still a developing field in many cancers (53); in case of myeloma patients, basing on the available literature, it’s a process that requires around 3-14 mL of blood to obtain ≧̸10^7^ cells per sample necessary to maintain NGF high sensitivity (54, 55), and offer a promising and minimally invasive alternative for tumor assessment, genetic characterization and extramedullary dissemination study of MM patients (56, 57). Flow-cytometry permits to detect CTCs easily, contributing in this way to understanding the pathogenesis of MM and to enlighten mechanisms of this disease that could be useful to clarify how other similar tumor develop and disseminate in the human body (57, 58).

## Bias of using NGF

Flow-cytometric analysis must be performed taking also in account some minor bias that could, if not considered, reduce the reliability of Multiple Myeloma MRD evaluations (24). First, often there is a high difference in terms of bone marrow cellularity and percentage of plasma cells observed by cytological analysis compared to flow-cytometry methods. This apparent inconsistency is due firstly to the fragility of plasma cells themselves, with a pool of plasma cells loss during laboratory manipulation; secondly, the lower PC count obtained by flow-cytometry may be related to a possible hemodilution of the BM samples, with the risk to underestimate the percentage of pathologic PCs (59). Different methods have been recommended to accurately evaluate the degree of hemodilution. These methods are based on an automated lymphocyte count, PB contamination indices that takes account of PC percentages, CD34^+^ cells, and CD10^+^ neutrophils (60), or numbers of CD16 bright neutrophils (61). In the case of flow cytometric analysis, NGF can also provide the qualitative assessment of patient samples by allowing for analysis of normal B-cell compartments and non-PC BM cells, such as mast cells or RBCs, which can give us a quite accurate estimation of the hemodilution of analyzed BM samples. Moreover, the good clinical practice of sparing the first aspirated sample from the iliac crest for flow-cytometric assays, could reduce the risk of performing MRD evaluation from low quality samples (62, 63).

The other major pitfall in MM MRD evaluation by NGF is correlated to the high heterogeneity of MM plasma cells phenotype, and to the possibility of a “shift” of plasma cells phenotype depending on the therapy that patients have been exposed (64, 65).. It has been widely demonstrated that patients starting treatment with immunomodulatory drugs may experience a change on plasma cells phenotype, and the use of drugs such as Daratumumab, that could mask the CD38 overexpressed molecule, can make even more difficult MM clones recognition (66). This last problem has been overcome by introducing CD38 multiepitope antibodies that permit, by binding to sites that are different from the one occupied by the drug, to still recognize MM PCs even during treatment with Anti-CD38 monoclonal antibodies (67). Moreover, the availability of analytic softwares like Infinicyt™ provides the possibility to analyze automatically MRD data and compare individual results to the set of data stored into database, increasing the accuracy and precision of the evaluation, and helping operators in those situations in which a manual gating could miss minor phenotypic alterations that could be related to resistance mechanisms or type of treatment (68). In combination, when possible, with NGS analysis, this approach could theoretically give the possibility to monitor adequately 100% of myeloma patients.

Finally, given the peculiarity of flow-cytometry analysis a high personnel expertise is essential in order to obtain reliable results, especially in demanding cases in which anti-CD38 therapy, presence of different pathologic clones or presence of normal PCs, together with low MRD burden, could lead to bias (69). Reducing the subjectivity in data analysis requires the work of experienced laboratories, that are constantly monitored and trained, and whose results could be assessed and tested by external quality assurance programs and interlaboratory comparisons.

## Discussion and future perspectives

Since MRD detection is now strongly recommended although not mandatory for guiding clinical treatment decisions, the possibility to employ NGF to test the depth and duration of response in Multiple Myeloma patients represents a great advantage and a great promise for the management of this disease. Additionally, NGF is an easy and low-cost technique and therefore is widely used nowadays for the analysis of MM MRD. The optimization of the quality of BM samples and the analytic expertise, that permits to discriminate properly the rare pathologic clones, are the main keys for obtaining results with a high clinical value that could be of great use in the future. The minor “foes” associated with the application of this technique could be easily overcome and do not reduce the value of using NGF for measurable residual disease of MM patients. With the use of NGF we can today enter a new phase of the quantification of residual disease, switching from the definition of “minimal” residual disease to “measurable” residual disease, and have the chance to accurately monitor MM patients and be able to early recognize those achieving long deep responses that may, in the future, be considered “cured” from the disease. Finally, the possibility to employ NGF for analysis of Circulating Tumor Cells (CTCs) represents a promising and minimally invasive alternative for tumor assessment and may enlighten mechanisms of disease dissemination that could be of great interest also applied to other cancers.

## Author contributions

Conceptualization, PP; methodology, PP, DR, and EB; validation, MB and AG; writing - original-draft preparation PP; writing-review and editing AG and MB. All authors have read and agreed to the published version of the manuscript.

## Funding

Funding institution is Azienda Ospedaliera Universitaria Senese, Siena, Italy.

## Conflict of interest

The authors declare that the research was conducted in the absence of any commercial or financial relationships that could be construed as a potential conflict of interest.

## Publisher’s note

All claims expressed in this article are solely those of the authors and do not necessarily represent those of their affiliated organizations, or those of the publisher, the editors and the reviewers. Any product that may be evaluated in this article, or claim that may be made by its manufacturer, is not guaranteed or endorsed by the publisher.
